# Exposed Rock Reduces Tree Size, but Not Diversity

**DOI:** 10.3389/fpls.2022.851781

**Published:** 2022-06-07

**Authors:** Jie Li, Lianjin Zhang, Yuanfa Li

**Affiliations:** ^1^College of Forestry, Guangxi Key Laboratory of Forest Ecology and Conservation, Guangxi University, Nanning, China; ^2^Experimental Center of Forestry in North China, Chinese Academy of Forestry, Beijing, China

**Keywords:** karst, diversity, habitat heterogeneity, stand structure, tree size, old-growth forest

## Abstract

Karst made up of limestone is widely considered a “Noah’s ark” of biodiversity. Rock and soil substrates comprise two different site types in karst terrain, although both can support dense forests. However, it is unclear whether and how the presence of exposed rock affects forest diversity and tree size. We established a 2.2 ha plot (200 × 110 m) in an old-growth oak forest (> 300 years) in karst terrain in southwestern China. We classified the plot into rock and soil components; we analyzed plant diversity and tree size in each component using species diversity indices (richness, number of individuals, Shannon–Wiener index, and Pielou evenness index), stand spatial structure parameters, diameter at breast height (DBH), tree height (TH), and tree basal area (BA). We also analyzed the distributional patterns of species at the sites using non-metric multidimensional scaling, then assessed the effects of abiotic environmental variables on diversity and tree size using redundancy analysis. Our results indicated that both site types (i.e., rock and soil) had similar overall species diversity; trees and shrubs were largely distributed at random within the study site. Tree size was evenly differentiated in the community, and trees were dominant, particularly on soil. Trees on rock were in a status of medium mixture, whereas shrubs on rock were highly mixed. The opposite trend was observed for trees and shrubs growing on soil. The DBH, TH, and BA were smaller in trees growing on rock than in trees growing on soil. Abiotic environmental variables had varying effects on the diversity and size of trees at the two site types; they only explained 21.76 and 14.30% of total variation, respectively. These results suggest that exposed rock has the effect of reducing tree size, but not diversity, thus highlighting the important role of rock in maintaining diversity; moreover, the results imply that karst microhabitats may mitigate the impacts of topography on tree diversity and growth. Greater attention should be focused on exposed rock in the conservation and management of karst forests and the restoration of degraded forest ecosystems.

## Introduction

Karst is a unique geological landform that results from the erosion and dissolution of bedrock *via* long-term hydrological processes (Du et al., 2013; Geekiyanage et al., 2018). Karst is extensively distributed worldwide; it occurs in numerous countries and regions (e.g., China, Vietnam, Thailand, Myanmar, Indonesia, the Mediterranean, and Brazil) encompassing a range of climatic zones (e.g., tropical, subtropical, temperate, and cold) in both island and continental regions (Clements et al., 2006; Ni et al., 2015; Geekiyanage et al., 2019; Li et al., 2019a; Gong et al., 2021; Zhang et al., 2022). The total global area of karst is 22,000,000 ha (i.e., 12–15% of the land surface; Wang et al., 2016; Zhu et al., 2017). Karst terrain is characterized by steep, irregular surfaces with frequent rock outcrops and thin, discontinuous soils, which create a complex mosaic of heterogeneous habitats at different scales (Crowther, 1987). Karst provides a suitable habitat for many wildlife species, including endangered species (Fitzsimons and Michael, 2017; Nie et al., 2018; Geekiyanage et al., 2019); it has been called a “Noah’s Ark” of biodiversity (Clements et al., 2006). Tropical and subtropical forests growing on karst terrain (hereafter, karst forests, or KFs) are often biodiversity hotspots (Zhang et al., 2012a; Do Carmo and Jacobi, 2015; Guo et al., 2021); they are ideal sites for biodiversity conservation, ecotourism, and explorations of the relationship between species and habitat complexity.

Heterogeneity in the abiotic environment has profound impacts on several aspects of KFs. Numerous studies have focused on the correlations between soil characteristics (e.g., nutrients, microorganisms, soil enzyme activity, organic matter, and pH) and the type (Du et al., 2013; Zhang et al., 2013; Zhu et al., 2017) and diversity (Zhang et al., 2012a; Do Carmo and Jacobi, 2015; Peng et al., 2020) of above-ground plant communities. They have also focused on the correlations between soil characteristics and non-spatial aspects of tree community structure (e.g., diameter at breast height, DBH; tree height, TH; basal area, BA; and crown width; Peng et al., 2012; Guo et al., 2016). Other studies have explored the relationships of topographic factors (e.g., slope, elevation, convexity, aspect, and degree of rock exposure) with species distribution, community composition, and biomass (Zhang et al., 2010, 2020a; Peng et al., 2012; Guo et al., 2017; Su et al., 2017). Moreover, some studies have compared the species compositions between sites on exposed rocks and sites on soil (Porembski et al., 1996, 1998; Nie et al., 2018). Up to now, however, there is a lack of consensus regarding the relationships between environmental variables and vegetation, which can be attributed to various factors including the complexity of karst terrain; the locality, interference, and degree of vegetation; and the characteristics of sampling (e.g., plot location, size, and shape) (Clements et al., 2006; Ni et al., 2015; Du et al., 2017; Nie et al., 2018). The effect of exposed rock, which is the key factor distinguishing karst and non-karst terrain, has been mentioned in numerous studies; it is rarely analyzed quantitatively, particularly at the quadrat scale.

Rock presumably acts as barrier in KF ecosystems, such that it affects surface and underground runoff from rainfall (Zhang et al., 2012b, 2020b), as well as the distribution of soil layers, soil water storage and utilization, soil nutrient and ion exchange (Wang et al., 2016; Zhu et al., 2017), and plant nutrient allocation (Zhao et al., 2020). These changes influence the growth processes (e.g., establishment, regeneration, competition, and mortality) and spatial patterns of tree species and communities (Fayolle et al., 2012; Simon et al., 2019). The survival and growth of some species depend on the degree of rock exposure (Ribeiro et al., 2007; Sadler and Bradfield, 2010). Exposed rock provides various microhabitats, including pits, crevices, gullies, surfaces, and walls. It exhibits high heterogeneity with respect to surface roughness and fracture size (Geekiyanage et al., 2019); it exerts strong environmental filtering in terms of the intensity of solar radiation, as well as water and nutrient availability (Gomes and Sobral-Leite, 2013), such that it is distinct from environments underlain by soil.

Exposed rock provides important habitat for both forbs and woody plants in humid climates (Felfili et al., 2007; Gomes and Sobral-Leite, 2013; Nie et al., 2018). Along with topography, it strengthens the connection between species and their abiotic environment; it substantially contributes to local biodiversity and biomass (Ni et al., 2015; do Carmo et al., 2016; Zhu et al., 2017). However, rupicolous ecosystems are fragile; they are sensitive to disturbances, such as land reclamation, fuelwood harvesting, burning, and grazing (Su et al., 2017). Vegetation restoration and reconstruction in disturbed regions become increasingly challenging (Zhang et al., 2010), which may lead to serious ecological and economic problems, as well as threats to the survival of local residents (Ni et al., 2015; Zhang et al., 2020b). Desertification in rock-dominated ecosystems has become a global issue. Fortunately, some old-growth and primary KFs have been preserved in remote mountainous, countryside, and island areas (e.g., southwest China and eastern Brazil; Liu et al., 2010; Gomes and Sobral-Leite, 2013; Zhang et al., 2013). These sites provide an ideal template for the management of degraded karst ecosystems (Zhang et al., 2013). Knowledge of the taxonomic and structural diversity of old-growth and primary KFs is limited; the role of rock in supporting, maintaining, and protecting diversity and tree growth remains poorly understood.

There is an either/or relationship between soil and rock in karst landscapes. Trees growing on rock lack a substrate to which they can attach, leading to a lack of nutrient and water sources. Resource limitations are likely to limit the diversity, development, and abundance of trees. Only xerophytic, barren-tolerant, and deeply rooted species, particularly shrubs, may be capable of adapting to such sites. We hypothesized that trees and shrubs growing on rock in old-growth KFs are smaller than trees and shrubs growing on soil, and that both species diversity and structural diversity are reduced in KFs (Hypothesis 1). Furthermore, the complex topography of karst terrain affects the spatial distribution of resources, such as light, heat, water, and soil nutrients. Therefore, we also hypothesized that karst terrain significantly affect patterns in tree size and diversity (Hypothesis 2).

## Materials and Methods

### Study Site

Our study site was in the Guangxi Yachang Orchid National Nature Reserve (106°11′ 31″–106°27′ 04″ E, 24°44′ 16″–24°53′ 58” N), in Leye County, Guangxi Zhuang Autonomous Region, China. The reserve extends 26.2 km from east to west and 18 km from north to south, with a total area of 22,062 km^2^. The site is within a mountainous area that forms the transition between the Yunnan–Guizhou Plateau and the Guangxi Hills. The Nanpan River, which forms the boundary between Guangxi and Guizhou provinces, flows through the area (Li et al., 2020). The dominant landforms in Guizhou province are large mountains composed of yellow brown soil, whereas the landscape in Guangxi is dominated by limestone. Substrates generally comprise either rock or a mixture of rock and soil; most areas are densely vegetated. Forest cover can reach 84.7%; it mainly consists of natural secondary forests of *Pinus yunnanensis* var. *tenuifolia* and oaks (*Quercus variabilis* Blume, *Quercus fabri* Hance), shrublands, and a small number of artificial forests. The region is characterized by a central subtropical monsoon climate; it is influenced by monsoon circulation and the foehn effect throughout the year. Humid oceanic air masses prevail in the summer (June–September), bringing high temperatures and rain. Cold continental air masses are dominant in the winter (December–February), whereas spring (March–May) and autumn (October, November) are characterized by severe drought. On average, the region receives 940.8–1,216.9 mm of rain per year and 1,303.7–1,698.7 h of sun. The mean annual temperature is approximately 16.3°C, but temperatures can reach highs of >40°C and lows of −3°C. Temperature and rainfall significantly vary with altitude; soil types also exhibit obvious vertical patterning, shifting from brown laterite in the valley to red and yellow soils on mountaintops. The soils are characterized by thin layers and high permeability, mineral content, and gravel content; they are generally barren (Li et al., 2020).

We established a study plot on a large mountain (106°23′ 12.6″ E, 24°49′ 55.3″ N) at the Huaping Nature Preservation Station. The upper boundary of the site coincided with the mountaintop and an east–west ridgeline. The lower boundary ran parallel to slope contours, whereas the left and right boundaries followed an altitudinal gradient. The plot had a mean elevation of 1,293 m and a mean slope of approximately 25°. The terrain was highly complex and provided diverse niches. Rock patches and individual rocks were exposed at the surface; they occupied a large portion of the plot and the surrounding area. The soil mainly comprised Rendzina and was rich in gravel (approximately 60%). The forest stand had been undisturbed for a long period of time; it was well-developed, with clear vertical stratification. Canopy cover was approximately 0.8, and the oldest tree (an oak) is estimated to be >300 years old. The forest may be one of the few old-growth KFs in both the region and in southwestern China. The canopy was dominated by *Q. variabilis* and *Q. fabri*; other common species included *Platycarya strobilacea* Sieb. & Zucc., *Keteleeria davidiana* (Bertr.) Beissn, *Rhus chinensis* Mill., *Betula alnoides* Buch.-Ham. ex D. Don, *Liquidambar formosana* Hance, and *Bothrocaryum controversum* (Hemsl.) Pojark. *Lyonia* species, including *Lyonia ovalifolia* (Wall.) Drude and *Lyonia villosa* (Wall. ex C. B. Clarke) Hand.-Mazz., dominated the shrub layer; other shrubs included *Viburnum cylindricum* Buch.-Ham. ex D. Don, *Callicarpa macrophylla* Vahl, and *Archidendron clypearia* (Jack) I. C. Nielsen. The understory supported abundant regeneration and was dominated by *Q. variabilis*, *Q*. *fabri*, *P. strobilacea*, and *Lyonia* spp. The herb layer was sparse and consisted of *Miscanthus floridulus* (Lab.) Warb. ex Schum. & Laut. and several small ferns. Lianas were rare; they were mainly represented by *Rubus alceaefolius* Poir., *Rubus coreanus* Miq., *Parthenocissus tricuspidata* (Siebold & Zucc.) Planch., and *Lonicera chrysantha* Turcz.

### Plot Establishment and Data Collection

**Figure 1:**
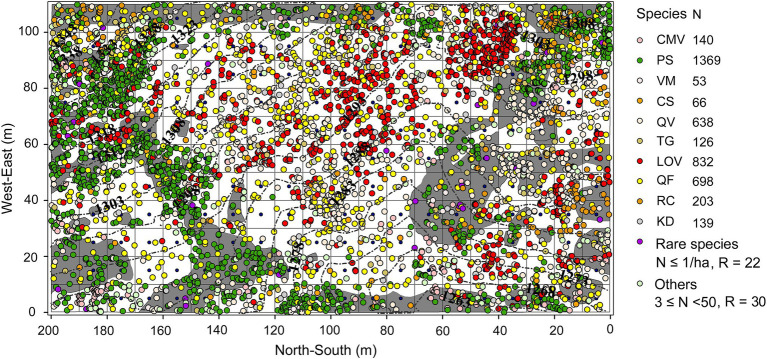
Species composition and distribution of rock and soil at the study site. Gray and white background colors represent rock and soil, respectively. The colored circles represent tree species, while the black dashed lines represent contours. CMV = *C. macrophylla*, PS = *P. strobilacea*, VM = *V. montana*, CS = *C. septentrionale*, QV = *Q. variabilis*, TG = *T. gymnanthera*, LOV = *L. ovalifolia*, QF = *Q. fabri*, RC = *R. chinensis*, KD = *K. davidiana.*

The establishment of quadrats *via* the traditional adjacent lattice method is difficult on karst terrain. Therefore, we used an improved method to establish a 200 m × 110 m fixed plot in mid-2019 in an old-growth KF on the upper slope of the mountain (Figure 1). We first used a total station (NTS-372R_10_, Southern Surveying and Mapping Company, Guangzhou, China) to establish the first boundary (length = 200 m) of the plot, which followed the slope contour; we then rotated the total station counterclockwise to establish the next boundary. We repeated this process two more times, thus forming a plot with a closure difference of less than 1/400. Next, we used the total station to subdivide the plot into 220 individual 10 m × 10 m quadrats (our ability to obtain coordinates was limited by the terrain). We inserted polyvinyl chloride pipes (*ø* = 6 cm) at the intersections of the quadrats and reinforced the pipes with steel rebar (*ø* = 1.2 cm). We then connected the pipes with plastic ropes to demarcate the boundary of each quadrat. We recorded the coordinates (*x*, *y*, *z*) of standing trees and deadwood (snags and fallen wood) with DBHs ≥1 cm using the “eccentric mode” of the total station. We measured the DBH (cm), crown width (m^2^), and TH (m) of standing trees and snags; we measured the length (m) and end diameters (cm) of fallen deadwood. We also recorded the species and growth status of standing trees (e.g., skew, dead branches, bends, broken shoots, and diseases) and the decay class (I–V) of fallen deadwood. Deadwood was identified to the species level based on buds, overall appearance (e.g., bark characteristics, size, and branching), and the species composition of adjacent trees. In addition, we marked each standing tree (DBH ≥ 5 cm) with numbered aluminum tags; we marked saplings (1 cm ≤ DBH < 5 cm) with numbered plastic plates. Finally, we sketched the location and outline of rock outcrops (surface area ≥ 0.2 m^2^) in each quadrat and determined the geographic coordinates and altitude (m) of each quadrat using a Global Positioning Systems (GPS) device. We recorded 4,596 live trees, 322 snags, and 33 pieces of fallen deadwood representing 62, 19, and 7 species, respectively. Of these, trees constituted 73.22% and shrubs constituted 26.78%. We documented 22 rare species (abundance = 1/ha; Table 1). Only data regarding standing trees were analyzed.

**Table 1:** Species composition of old-growth oak forest located at karst terrain.

Species	Abbreviation	*N*	MTH	MDBH	Life form	Soil/Rock
*P. strobilacea*	PS	1,369	4.50	2.94	Tree	+/+
*L. ovalifolia*	LOV	832	3.29	2.14	Shrub	+/+
*Q. fabri*	QF	698	14.72	24.83	Tree	+/+
*Q. variabilis*	QV	638	6.85	8.48	Tree	+/+
*R. chinensis*	RC	203	5.04	3.53	Shrub	+/+
*Callicarpa macrophylla Vahl*	CMV	140	4.10	2.23	Shrub	+/+
*K. davidiana*	KD	139	5.99	7.34	Tree	+/+
*Ternstroemia gymnanthera* (Wight et Arn.) Sprague	TG	126	4.19	3.33	Tree	+/+
*Cinnamomum septentrionale* Hand.-Mazz	CS	66	4.28	3.96	Tree	+/+
*Vernicia montana* Lour.	VM	53	2.81	2.21	Tree	+/+
*B. alnoides*	BA	42	5.85	4.50	Tree	+/+
*Myrica rubra* (*Lour.*) *S. et* Zucc.	MR	30	4.44	5.59	Shrub	+/+
*Albizia kalkora*	AK	23	3.97	2.69	Shrub	+/+
*Lyonia villosa* (Wall. ex C. B. Clarke) Hand.-Mazz.	LV	21	3.01	1.79	Shrub	+/+
*Populus* L.	PL	18	3.91	2.35	Tree	+/+
*Ailanthus altissima* (Mill.) Swingle	AA	17	11.80	12.01	Tree	+/+
*L. formosana*	LF	15	9.12	7.87	Tree	+/+
*Litsea pungens* Hemsl.	LP	14	5.14	5.12	Tree	+/+
*Camptotheca acuminata.*	CA	13	5.73	3.86	Tree	+/+
*Mahonia fortunei* (Lindl.) Fedde	MF	11	2.84	1.78	Shrub	+/+
*Broussonetia kazinoki* S. et Z.	BK	10	4.45	3.23	Shrub	+/+
*Prunus tomentosa*	PT	6	4.03	3.89	Shrub	+/+
*Lindera nacusua* (D. Don) Merr.	LN	6	5.52	4.09	Shrub	+/+
*Pinus yunnanensis* Franch. *var. tenuifolia*	PY	5	9.24	19.69	Tree	+/+
*Coriaria nepalensis* Wall.	CN	5	4.20	4.59	Shrub	+/+
*Kalopanax septemlobus* (Thunb.) Koidz.	KS	5	8.58	9.83	Tree	+/+
*Glochidion puberum* (L.) Hutch.	GP	5	5.14	2.68	Shrub	+/+
*Clerodendrum mandarinorum* Diels	CM	5	5.20	4.85	Shrub	+/+
*Fraxinus insularis* Hemsl.	FI	5	4.26	2.21	Tree	+/+
*Lyonia ovalifolia* (Wall.) Drude *var.*	LO	4	2.28	2.01	Shrub	+/−
*Chionanthus ramiflorus Roxburgh*	CR	4	4.70	4.53	Shrub	−/+
*Cinnamomum camphora* (L.) *Presl*	CC	3	6.80	6.53	Tree	+/+
*Cinnamomum glanduliferum* (Wall.) *Nees*	CG	3	4.30	2.56	Tree	+/+
*Eriobotrya japonica* (Thunb.) Lindl.	EJ	3	2.27	1.21	Tree	−/+
*lniphyllum fortunei* (Hemsl.) *Makino*	IF	3	2.83	1.53	Tree	+/+
*Cladrastis platycarpa* (Maxim.) *Makino*	CP	3	3.77	1.95	Tree	+/+
*Acer davidii Franch.*	AD	3	4.83	3.00	Tree	+/+
*Ilex micrococca* Maxim.	IM	9	5.37	3.38	Shrub	+/+
Unknown	H	7	7.39	6.91	Tree	+/+
*Sinoadina racemosa* (Sieb. et Zucc.) Ridsd.	SR	6	10.18	5.77	Tree	+/+
Rare species		28	5.8	5.1		

‘+’ = occurring, ‘−’ = missing, *N* = abundance, MTH = mean tree height (m), MDBH = mean diameter at breast height (cm).

### Data Analyses

#### Extraction of Topographic Factors

We imported the coordinate data into ArcGIS 10.2^1^ to create a digital elevation model of the plot, which we used to extract topographic information (i.e., elevation, convexity, aspect, and slope) for each quadrat. The mean value of the four vertices was used to represent quadrat elevation (m). Convexity was quantified by subtracting the mean elevation of the quadrat from the elevation of the quadrat center. Positive and negative values indicate that the center is higher or lower than the surrounding area, respectively; zero indicates little variation in elevation. Slope (°) was represented by the mean angle of the four planes formed by any three vertices in the quadrat. Aspect was represented by the mean value of the angle between true north and the orientation of the four planes comprising three vertices. We also scanned the field sketches and aligned them to the topographic map; subsequently, we outlined rock and soil patches, then calculated the area of each (6,224 and 15,776 m^2^, respectively). To determine whether rock affected taxonomic and structural diversity or tree size, we defined rock quadrats as quadrats in which rock constituted ≥50% of the substrate. Quadrats with <50% rock cover were considered soil quadrats. In total, 61 rock quadrats (6,100 m^2^) and 159 soil quadrats (15,900 m^2^) were surveyed.

#### Taxonomic and Structural Diversity and Tree Size

Taxonomic (species) and structural diversity are key components of forest diversity. We used four traditional species diversity indices (i.e., richness, abundance, the Shannon–Wiener index [*H*′], and the Pielou evenness index [*E*_H_]) to describe the diversity of tree species at the plot and quadrat scales (Table 2). These indices are widely used in ecology and forestry; they have been explored in great detail (Li et al., 2021). Diversity indices were calculated using the *diversity* function in the *vegan* package (Oksanen et al., 2019) in R.^2^ We analyzed the spatial relationship between a reference tree *i* and its four nearest neighbors using a set of stand spatial structure parameters, including the uniform angle index, dominance, and mingling; these represent relative spatial position, size differentiation, and species mixture, respectively (Table 2). The advantage of stand spatial structure parameters is that they allow the calculation of three-parameter values for each tree based on explicit biological information. They may also be expressed in various ways, including mean values and univariate-, bivariate-, and trivariate distributions (Hui et al., 2019; Li et al., 2020, 2021; Zhang and Hui, 2021). We calculated parameter values for each tree in the stand and mean values for each quadrat, as well as the parameter values for all trees and shrubs occurring on rock and soil, respectively. In addition, we calculated tree size indicators for each quadrat, including mean TH, DBH, and BA.

**Table 2:** Species diversity indices and stand spatial structure parameters used in this study.

	Formula	Explanation	References
SDI	$R=\overset{s}{\underset{i=1}{\sum }}1$	*R* = richness, *S* = number of species.	any statistical book
	$N=\overset{s}{\underset{i=1}{\sum }}n_{i}$	*N* = all trees, abundance; *n_i_* = number of tree in species *i*.	any statistical book
	$H'=-\overset{s}{\underset{i=1}{\sum }}p_{i}\ln\left(p_{i}\right)$	$H^{\prime }$ = Shannon–Wiener index, *S* = number of species, *p_i_* = proportion of individuals in the *i*th species.	Li et al., 2020, 2021
	$E_{H}=\frac{-\sum p_{i}\log p_{i}}{\ln S}$	$E_{H}$ = Pielou evenness index, *S* = number of species, *p_i_* = proportion of individuals in the *i*th species.	Li et al., 2020, 2021
SSSPs	$W_{i}=\frac{1}{4}\overset{4}{\underset{j=1}{\sum }}Z_{ij}$	*W* = Uniform angle index; when the *j*th angle α is smaller than the *i*th standard angle $\alpha_{0}$ , *z_ij_* is equal to one. Or, *z_ij_* is 0.	Hui et al., 2019 Li et al., 2020, 2021
	$U_{i}=\frac{1}{4}\overset{4}{\underset{j=1}{\sum }}K_{ij}$	*U* = Dominance; when the reference tree *i* is smaller than the neighbor tree *j*, *k_ij_* is equal to one. Or, *k_ij_* is 0.	Hui et al., 2019 Li et al., 2020, 2021
	$M_{i}=\frac{1}{4}\overset{4}{\underset{j=1}{\sum }}V_{ij}$	*M* = Mingling; when the neighbor *j* is not the same species as the reference tree *i*, *v_ij_* is equal to one. Or, *v_ij_* is 0.	Hui et al., 2019 Li et al., 2020, 2021

### Relationship Between Habitat and Diversity

We considered rock and soil to be distinct site types; we estimated the linear relationships between the topographic attributes of each type and the mean values of the diversity (richness, abundance, *H*′, *E*_H_), structural, and tree size (DBH, TH, BA) indices. We then explored differences between site types using the *Kruskal*.*test* function. Furthermore, we considered the area of rock and soil and the four topographic attributes (slope, aspect, convexity, and elevation) as indicators of the abiotic environment; we used redundancy analysis to determine the degree to which these abiotic variables explained variations in species distributions, structural diversity, and tree size. The *envfit* function in the *vegan* package was used to test the significance of each variable. We applied non-metric multidimensional scaling to analyze the distributions of tree species on rock and soil using the *rad* and *metaMDS* functions in the *vegan* package (Oksanen et al., 2019), respectively. In addition, we analyzed the univariate distributions of stand spatial structure parameters for trees and shrubs growing in the two site types, then tested their similarity using the *ks*.*test* function.

## Results

### Species Diversity on Rock and Soil

**Figure 2:**
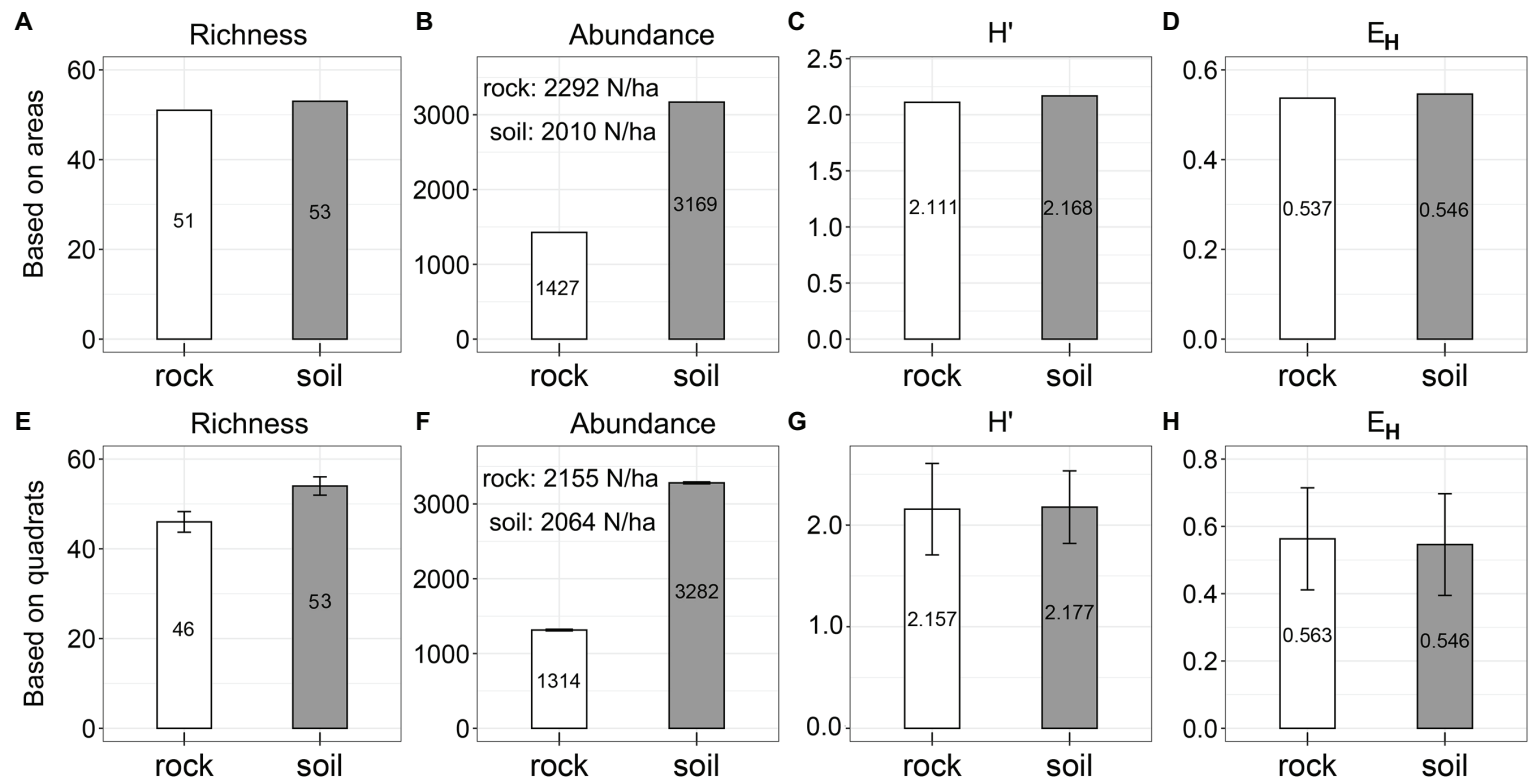
Species diversity on rock and soil. *H*′ = ShannonWiener index, and *E*_H_ = Pielou evenness index.

Species richness was similar between rock (53 spp.) and soil (51 spp.; Figure 2A; *p_kw_* = 0.275). Soil had seven more spp. than rock if counted by quadrat (Figure 2E). We detected significantly more trees on soil (3,169) than on rock (1,427, Figure 2B; *p_kw_* = 0.044). However, abundance on a per-hectare basis was very similar between rock and soil, based on both the actual area and the number of quadrats of each site type (rock: abundance = 2,010–2,292/ha, Figure 2B; soil: abundance = 2,064–2,155/ha, Figure 2F). Values for *H*′ (rock: 2.111–2.157; soil: 2.168–2.177; *p_kw_* = 0.575) and *E*_H_ (rock: 0.537–0.563; soil: 0.546–0.546) were also very similar (Figures 2C,D,G,H).

**Figure 3:**
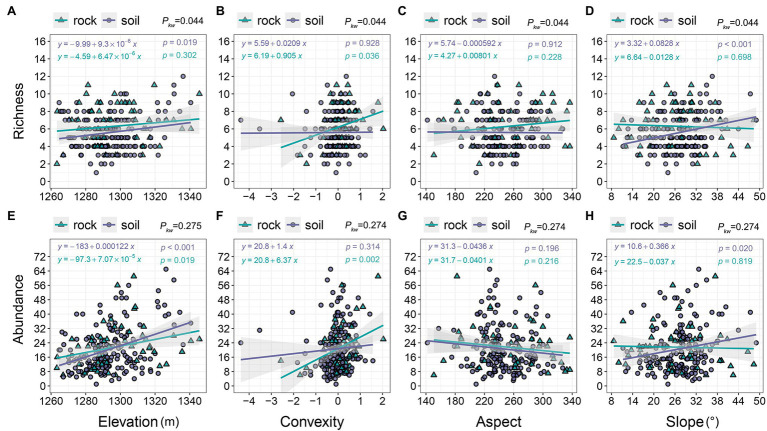
The relationships of richness and abundance with topographic variables. *p_kw_* > 0.05, 0.01 < *p_kw_ <* 0.05, and *p_kw_* < 0.01 represent non-significant, significant, and highly significant differences, respectively.

The richness and abundance of both site types increased along an elevation gradient (1260–1,350 m; Figures 3A,E), but richness was higher on rock than on soil (Figure 3A). Increases in convexity from −4 to 2 corresponded to rapid increases in richness and abundance on rock (2–11 and 6–61, respectively), but they only corresponded to minor increases on soil (Figures 3B,F). We observed decreases in richness as the aspect increased from 140 to 340 (Figure 3G), whereas richness varied little on soil but exhibited a slight increase on rock (Figure 3C). The richness and abundance of soil increased slightly with increasing slope (8–50°), whereas the richness and abundance of rock exhibited a slight decline with increasing slope (Figures 3D,H).

**Figure 4:**
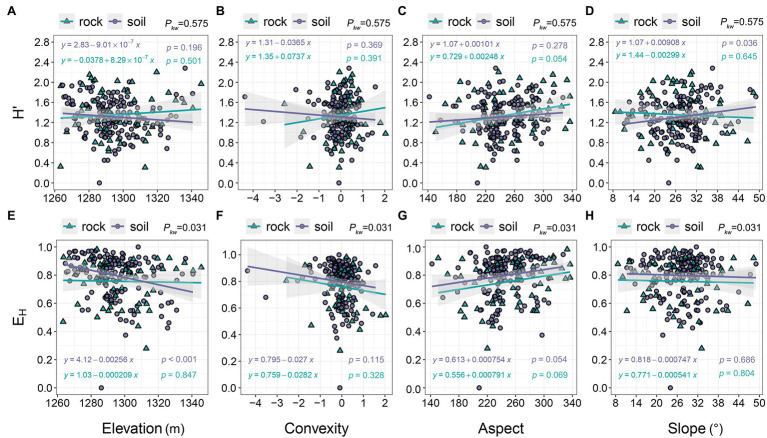
The relationships of *H*′ and *E*_H_ with topographic variables. *p_kw_* > 0.05, 0.01 < *p_kw_ <* 0.05, and *p_kw_* < 0.01 represent non-significant, significant, and highly significant differences, respectively. *H*′ = ShannonWiener index, and *E*_H_ = Pielou evenness index.

The *H*′ of communities on rock exhibited a slight increase with increasing elevation (0.307–2.202), while the *H*′ of communities on soil exhibited the opposite trend (Figure 4A). Decreases in *E*_H_ were more pronounced in communities on soil than in communities on rock (Figure 4E). E_H_ decreased as convexity increased (Figure 4F), as did the *H*′ of communities on soil (0.451–2.281), whereas the *H*′ of communities on rock increased (Figure 4B). *H*′ and *E*_H_ increased gradually with aspect (Figures 4C,G). *E*_H_ decreased as slope increased (Figure 4H). The *H*′ of communities on rock also decreased with slope, whereas the *H*′ of communities on soil gradually increased (Figure 4D).

### Stand Spatial Structure Parameters of Communities on Rock and Soil

**Figure 5:**
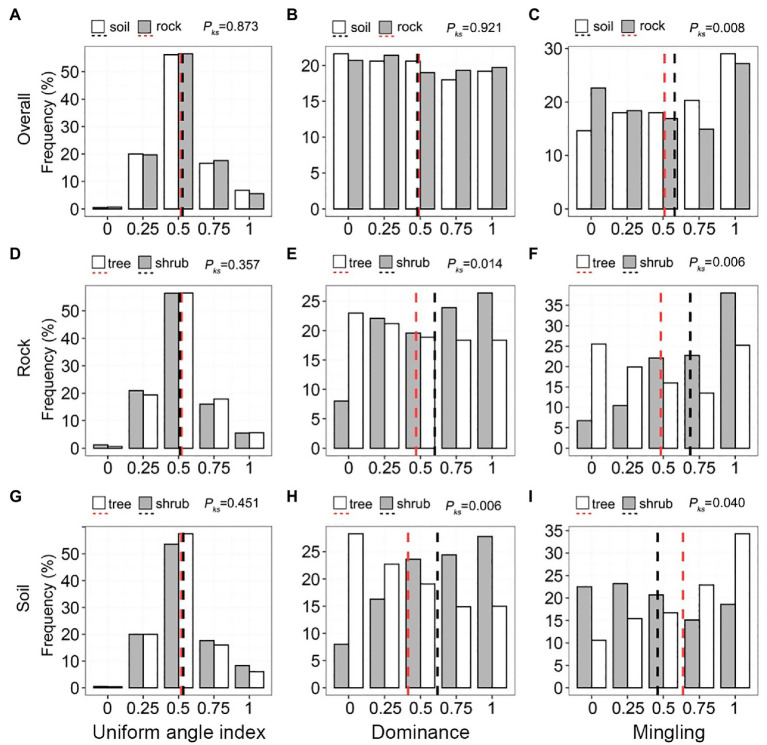
Univariate distributions of the stand spatial structure parameters of trees and shrubs on rock and soil. The red and black dashed lines represent the mean values of each parameter. *p_ks_* > 0.05, 0.01 < *p_ks_ <* 0.05, and *p_ks_* < 0.01 represent non-significant, significant, and highly significant differences, respectively.

The uniform angle index of communities on rock and soil exhibited a unimodal pattern (Figure 5A), with mean values (0.519 and 0.523, respectively) similar to the values of a random distribution (0.475–0.517). The distribution and mean values of uniform angle index (0.482–0.489) were also very similar (Figure 5B; *p_ks_* = 0.921). Most individuals (50.62–57.93%) were in a state of low–medium mixture (*M* = 0.00–0.50); the mean mingling of communities on rock (0.514) was significantly lower than the mean mingling of communities on soil (0.578; Figure 5C). The uniform angle index of trees and shrubs growing on rock and soil were very similar to the uniform angle index of trees and shrubs in the forest stand (Figures 5D,G). Higher values of dominance corresponded to increased shrub frequency and decreased tree frequency (Figures 5E,H). Shrub frequency on rocks (6.75–38.04%) increased gradually with increasing mingling, whereas tree frequency exhibited a decreasing trend (Figure 5F). The mingling of shrubs growing on soil did not significantly vary (15.08–23.17%), whereas the mingling of trees exhibited a gradual increase (Figure 5I).

**Figure 6:**
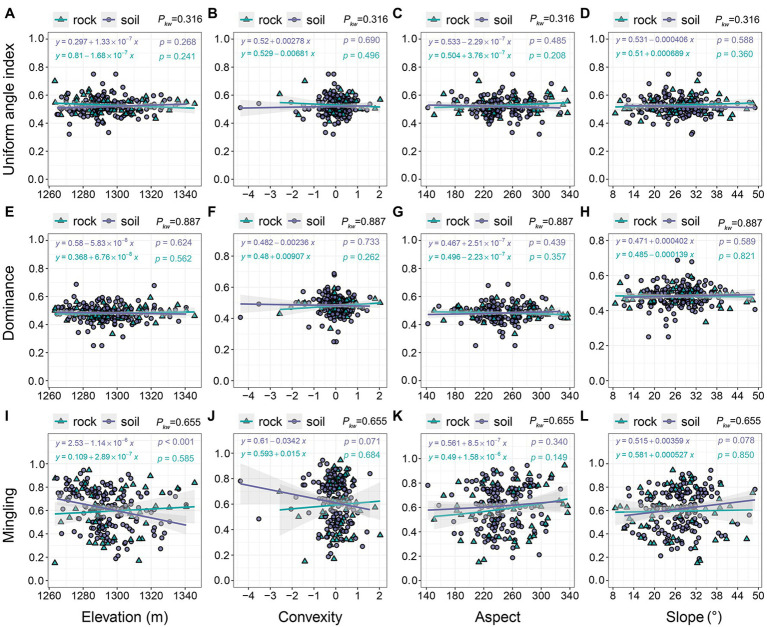
The relationships of stand spatial structure parameters with topographic variables. *p_kw_* > 0.05, 0.01 < *p_kw_ <* 0.05, and *p_kw_* < 0.01 represent non-significant, significant, and highly significant differences, respectively.

Topographic variables had little effect on uniform angle index or dominance: the models showed a clear, horizontal trend in which values of uniform angle index and dominance remain nearly constant at approximately 0.5 (Figures 6A–H). We observed no significant differences in uniform angle index or dominance between communities on rock and soil (*p_kw_* = 0.316–0.887). Topographic variables also had little influence on mingling (Figures 6I–L). The mingling of trees growing on soil, which ranged from 0.188 to 0.944, decreased with increasing elevation and convexity (Figures 6I,J); however, it slightly increased with increasing aspect and slope (Figures 6K,L). The mingling of species growing on rock exhibited slight increases in response to all four topographic variables (0.150–0.944; Figures 6I–L).

### Tree Size on Rock and Soil

**Figure 7:**
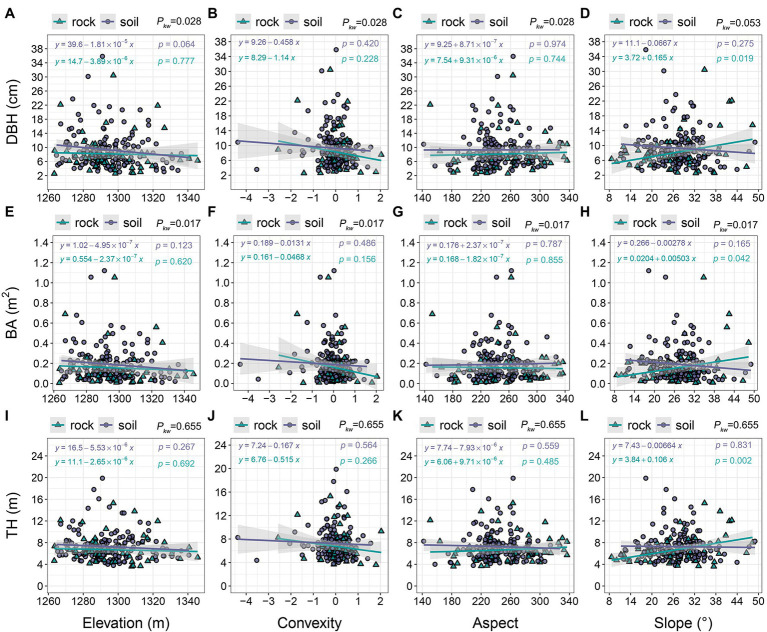
The relationships of tree size with topographic variables. *p_kw_* > 0.05, 0.01 < *p_kw_ <* 0.05, and *p_kw_* < 0.01 represent non-significant, significant, and highly significant differences, respectively. *DBH* = diameter at breast height, *BA* = basal area, and *TH* = tree height.

Tree size slightly decreased with increasing elevation in both site types. Linear models indicated that trees growing on soil were larger than trees growing on rock at most elevations (1260–1,325 m; Figures 7A,E,I); there were significant differences in DBH and BA (*p*_kw_ = 0.017–0.028). Tree size decreased with increasing convexity, and the decrease was more apparent in trees growing on rock than in trees growing on soil (Figures 7B,F,J). Changes in tree size among aspects were minor, but trees growing on soil were generally larger than trees growing on rock (Figures 7C,G,K). The size of trees growing on rock markedly increased with increasing slope (DBH = 2.54–30.44 cm; BA = 0.006–1.053 m^2^; TH = 3.60–15.28 m), whereas the size of trees on soil exhibited a decreasing trend (Figures 7D,H,L). TH did not significantly differ between the two site types (*p*_kw_ = 0.655).

### The Influence of the Abiotic Environment on Diversity and Tree Size

**Figure 8:**
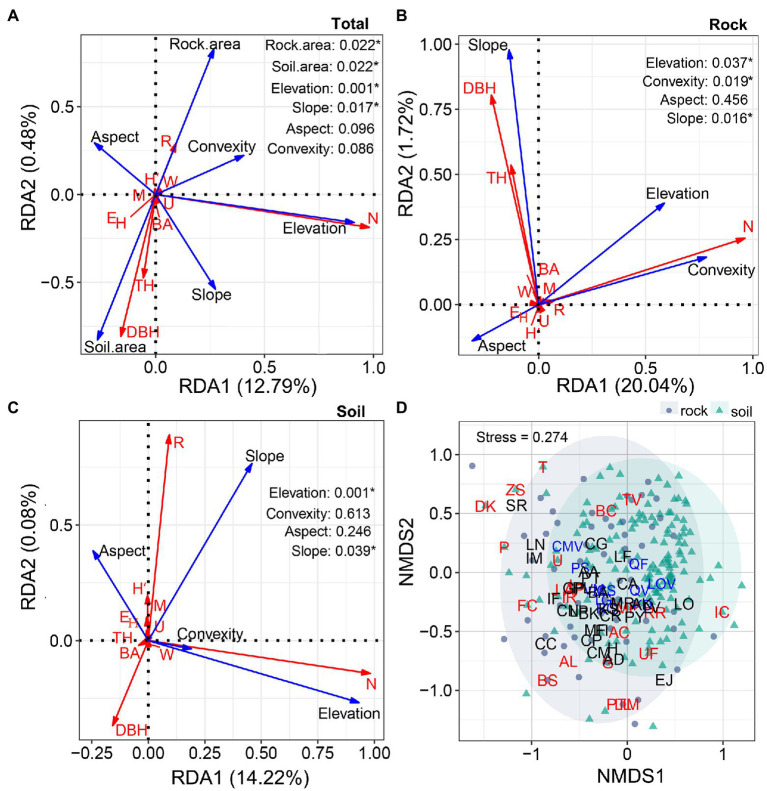
Redundancy analysis ordinations illustrating the influences of environmental variables (area of soil and rock, elevation, convexity, aspect, and slope, represented by blue arrows) on diversity (richness (R), abundance (N), *H*′, *E*_H_, uniform angle index (W), mingling (M), and dominance (U), represented by red arrows) and tree size (TH, DBH, and BA, represented by red arrows; **A–C)**. Non-metric multidimensional scaling ordinations of compositional patterns in the rock and soil quadrats **(D)**. Blue letters represent the 10 most abundant species, red letters represent rare species (*N* ≤ 1/ha; *R* = 22), and black letters represent other species (3 ≤ N < 50; *R* = 30). The solid gray circles represent rock quadrats, and the cyan triangles represent soil quadrats.

At the quadrat scale, four environmental variables (area of rock and soil, elevation, and slope) significantly affected the distributions of species diversity and tree size (*p* < 0.05). The other two environmental factors (convexity and aspect) had little effect (*p* > 0.05). The environmental variables explained 13.27% of total variation (RDA1 = 12.79%; RDA2 = 0.48%; Figure 8A). With respect to site type, slope was positively correlated with the DBH and TH of trees growing on rock, whereas elevation and convexity were positively correlated with the abundance of trees (Figure 8B). Slope was positively correlated with the richness and negatively correlated with the DBH of trees growing on soil. Elevation was positively correlated with the abundance of trees growing on soil (Figure 8C). Of the environmental variables considered, the area of rock explained 21.76% of total variation, while the area of soil explained 14.3%. Most species were clumped, with some dominant species (e.g., *P. strobilacea* and *R. chinensis*) concentrated on rock, and others (e.g., *R. chinensis*, *Q. variabilis*, and *Q. fabri*) concentrated on soil. The 22 rare species occurred primarily in the rock quadrats; the remaining species were evenly distributed between the two site types.

## Discussion

### The Relationship Between Karst Habitats and Species Diversity

Areas underlain by rock and soil represent two fundamentally different site types in karst terrain, but their plant communities were similar in terms of richness and abundance per unit area (Figures 2A,B,E,F). These findings demonstrate that rock does not reduce plant density or richness; moreover, rock has a crucial role in the maintenance of species diversity in old-growth KFs. Many species in karst habitats exhibit a set of mechanisms that are adaptive within particular environments. For example, species may be rupicolous, or exhibit drought tolerance (with respect to photosynthetic performance, xylem hydraulic characteristics, osmotic regulation, antioxidant enzymes, or leaf structure (Liu et al., 2010; Vilhar et al., 2010; Geekiyanage et al., 2018; Zhang et al., 2020a), barren tolerance (Peng et al., 2012; Zhu et al., 2017), leaf shed during the dry season (Felfili et al., 2007), reduced growth or dwarfing (Felfili et al., 2007; Liu et al., 2018; Zhang et al., 2020b), or improved root–shoot ratios (Ni et al., 2015). We also found that some species (e.g., *P. strobilacea* and *R. chinensis*) maintain their dominance and increase their probability of regeneration through high seed production. Patterns of species richness and abundance in karst landscapes may be the result of long-term processes. Vegetation establishes faster and more readily on soil than on rock (Nie et al., 2018). This helps to improve the microhabitats found on exposed rock, presumably through mechanisms that include generating vegetative cover and trapping litter to form humus and soil (Ni et al., 2015; Zhu et al., 2017); it also provides some provenances, promotes subsequent species establishment, and results in a pattern of multi-species coexistence in late succession. In addition, the abundance patterns of dominant species markedly differed between sites (Figure 1), suggesting that species exhibit habitat preferences and are more abundant in their preferred sites (Zhang et al., 2010).

Topography is an important source of habitat heterogeneity at small and medium scales; it indirectly influences species composition, abundance, and distributions. Several studies have demonstrated that altitude is the most important topographic factor in subtropical primary KFs (Zhang et al., 2007, 2010; Peng et al., 2012). Increasing elevation corresponds to increased rainfall, light, heat, and wind speeds, which accelerate the decomposition of rocks and increase their surface roughness (do Carmo et al., 2016); these changes promote the growth of light- and drought-tolerant species (i.e., most species at our study site) and increase their abundances. We found that species richness and abundance on rock and soil increased with increasing elevation (Figures 3A,E), indicating that most species prefer to grow on mountaintops. Similar phenomena have been observed in other primary and mature KFs in adjacent regions (Zhang et al., 2010; Du et al., 2017; Guo et al., 2019). Specialized karst habitats can support more species (Clements et al., 2006; Do Carmo and Jacobi, 2015; Du et al., 2017). In contrast, low-altitude areas are susceptible to seasonal waterlogging, flooding, erosion, shading by large trees, and rockslides, which may restrict the number of species that can persist in these habitats (Geekiyanage et al., 2018; Guo et al., 2019). Species growing on soil occurred preferentially on convex surfaces (Figures 3B,F, 8B), southwest-facing slopes (Figures 3C,G), and steep slopes (Figures 3D,H), further underlining the habitat preferences exhibited by species that grow in karst terrain.

*H*′ and *E*_H_ were nearly identical among the two site types (Figures 2C,D,G,H), indicating that rock does not reduce species diversity; this finding contradicts our first hypothesis. The increased *H*′ on rock is attributable to the presence of rare species at high altitudes (Figure 4A), which provides additional evidence that the complex niches found in rock outcrops promote higher species diversity (Zhang et al., 2010; Speziale and Ezcurra, 2012; Wang et al., 2016). Soil provides comparatively simple microhabitats, such that species are more strongly influenced by topography. Increased elevation supported greater proportions of some dominant species (i.e., *L. ovalifolia* and *Q. fabri*), as well as greater imbalances in interspecific abundance (Stein et al., 2014); these changes resulted in decreased *E*_H_ (Figure 4E). Aspect influences the duration and intensity of light to which plant communities are exposed. While our study site encompassed a diversity of aspects (Figures 3, 4, 6, 7), these had little effect on *H*′ or *E*_H_. This may be related to the high light conditions characteristic of subtropical mountaintops, and the resulting lack of differences in light conditions among quadrats with different aspects (Guo et al., 2016). Both slope and concavity are closely related to site fertility and moisture levels (Guo et al., 2016), although they weakly influence these two indicators (Figures 4B,D,F,H). Thus, topographic factors exhibited distinct effects on the *H*′ and *E*_H_ of communities growing on soil and rock. There are few relevant studies on karst, and no consensus has been reached (Peng et al., 2012; Du et al., 2013; Zhang et al., 2013; Guo et al., 2016), reflecting the complexity and species diversity of karst habitats.

### The Relationship Between Karst Habitats and Stand Structural Diversity

The spatial structural diversity of forest communities is attracting increasing attention (Li et al., 2020; Zhang and Hui, 2021). The distributions of uniform angle index values of the trees and shrubs at our site are consistent with the distributions of uniform angle index values in natural forests that have remained undisturbed for a long period of time (Li et al., 2014, 2020; Zhang et al., 2018; Yang et al., 2019). They were largely distributed at random (Figure 5), indicating that site and lifeform have little influence on distributional patterns. The proportion of random trees (*Wi* = 0.50) to the total number of plants determined the type of distributional pattern. Recent studies have reported that random trees are the cornerstones of non-karst natural forests; they have no relationship to forest type, geographical distribution, species composition, diameter, canopy crowding, competition, or tree point patterns (Zhang et al., 2018; Zhang and Hui, 2021), strongly supporting our results. Li et al. (2020) also found that tree size was unrelated to distributional patterns in a neighboring mixed forest. However, at very small minimum tree diameters, the proportion of random trees may substantially diminish, whereas the proportion of clustered trees increases (Li et al., 2019b). Although we do not know the specific conditions for the development of random distributional patterns in KFs, such patterns are undoubtedly the result of the long-term interspecific, intraspecific, and species–environment interactions (Guo et al., 2017). Our findings validate the “random structural framework stability” hypothesis with respect to site and life form (Hui et al., 2021).

The size differentiation of neighboring trees in the old-growth KF was balanced (Figure 5B), such that trees of different sizes were randomly distributed throughout the plot. This is a common feature of many non-karst natural forests (Li et al., 2014, 2020; Zhang et al., 2018; Yang et al., 2019). Trees always occupy a dominant position in areas underlain by soil (Figures 5E,H), which indicates that rock inhibits development and reduces competition between adjacent trees. It also implies that the relationships of lifeforms are unequal, which is consistent with our first hypothesis. Species mixture may reflect the adaptative strategies of species to habitats underlain by rock versus soil. The high degree of species mixture in communities growing on soil (Figure 5C) reduced conspecific competition and mortality, while improving species diversity and the survival rates of weaker competitors (Raventós et al., 2010). The low degree of mixture observed on rock (Figure 5C) helps to reduce adversity (Medina et al., 2006) and improves the probability of survival. Intraspecific aggregation is an important characteristic of species’ spatial distributions in KFs (Zhang et al., 2010; Guo et al., 2017; Lu et al., 2021); it is common in communities growing in harsh habitats (e.g., xeric, halomorphic, or alpine; Callaway et al., 2002; Guo et al., 2021). Deadwood is uncommon on rock, suggesting that intraspecific associations are typically facilitative, rather than competitive. Surprisingly, topography had only a weak effect on spatial structural diversity (Figures 6A–L, 8A–C). In addition to the spatial architecture and rocks typical of karst terrain, some ecological processes unique to karst (e.g., its dualistic hydrological structure; Geekiyanage et al., 2019), as well as its complex physical structure and the scale of sampling, may also reduce the explanatory power of these variables (Du et al., 2013, 2017; Wang et al., 2016; Guo et al., 2017). Furthermore, positive correlations among topographic factors may reduce their interpretability (Figures 8B,C). To our knowledge, few studies have investigated the relationship between spatial structural diversity and habitat.

### The Relationship Between Karst Habitats and Tree Size

Trees growing on rock were smaller than trees growing on soil (Figure 7), which indicates that growth is reduced or delayed on rock, consistent with size differentiation and our first hypothesis. Other studies have also reported that trees growing on rock are slenderer and have lower biomass than do trees growing on soil or in nearby, non-karst forests (Ni et al., 2015; do Carmo et al., 2016). While rock is rich in a small number of nutrients (e.g., calcium and magnesium), it is insufficient in most others (Du et al., 2013; Zhang et al., 2013). Nutrient availability is the main factor that restricts the growth of trees in karst terrain (Huang et al., 2008; Peng et al., 2012; Fitzsimons and Michael, 2017). Increased area of exposed rock is associated with lower resource availability, leading to smaller trees (Figure 8A). Conversely, increased soil cover is associated with adequate nutrient sources, thus improving growth (Figure 8A). Most species occurred on both rock and soil (Table 1), but some dominant species were in different life stages in the two site types, resulting in size differences. For example, many individuals of the dominant species in the canopy (e.g., *Q. variabilis* and *Q. fabri*) and understory (*L. ovalifolia*) were over-mature on soil; in contrast, individuals growing on rock were reaching maturity during the study period and most (e.g., *P. strobilacea* and *R. chinensis*) were young trees. The diameter distributions of primary and secondary KFs reportedly exhibit an inverted J shape (Zhang et al., 2012a; Liu et al., 2018), but the previous studies did not consider the effect of site differences on the distributions of trees of various sizes. In conclusion, species composition and tree size on rock may change with succession, and rock contributed to growth differences at our study site.

Steeper slopes are associated with smaller trees (Figure 8C). Substrate, nutrients, and soil water are easily lost from steep slopes because of runoff; they accumulate in low-lying areas, changing the spatial patterns of plant nutrient availability, thus affecting spatial patterns in tree size (Zhang et al., 2013; Do Carmo and Jacobi, 2015; Guo et al., 2017; Geekiyanage et al., 2018). This trend is illustrated by large *L. formosana* occurrence in the lowlands and *L. ovalifolia* occurrence at higher elevations. Patterns in tree size distribution have been extensively explained from an ecophysiological perspective (Liu et al., 2010; Geekiyanage et al., 2018). Species on steep, rocky slopes are larger than species in other rocky areas (Figure 8B). These slopes are prone to water erosion, creating additional textures and crevices that encourage the growth of rupicolous species. The effect of rocky microhabitats on tree growth should clearly not be ignored. Larger trees exhibit higher biomass and higher BA. Guo et al. (2019) reported higher BA on steep slopes in the Nonggang karst plot (Guangxi, China), but they did not consider site type. The other three terrain factors (elevation, convexity, and aspect) had weak relationships with tree size (Figures 8B,C). The effects of topographic factors on tree growth are often inconsistent (Guo et al., 2016). Karst rocks are themselves highly variable and haphazard (Geekiyanage et al., 2019), a characteristic that influences species composition, distribution, and growth at multiple scales.

## Conclusion

Rock may be the greatest obstacle to the restoration of degraded karst ecosystems. Rock underlies a large amount of woodland; it also hinders the establishment, growth, and species associations of trees. However, numerous woody plants occur on rock and soil in old-growth and primary KFs. We found that rock and soil were equally important for promoting diversity, although the underlying mechanisms differed. Rock increases species diversity by providing more microhabitats, thus promoting increased numbers of rare species. Rare species on rock are scattered; the common species on rock are smaller and less abundant. They were clumped, exhibited high regeneration, and were better adapted to utilizing the rock habitat. In contrast, individuals growing on soil were larger, with smaller differences in interspecific abundance and higher species mixture, similar to the structural characteristics of mature non-karst forests. Species growing on soil may contribute more to biomass accumulation and carbon sinks. Notably, we found that trees occur preferentially on rock, whereas shrubs occur preferentially on soil. Trees are presumably able to grow roots deep into the rocks; this allows them to absorb water and nutrients, while increasing their structural stability. Conversely, shrubs can only access water and nutrients from the topsoil. This reflects the different strategies of trees and shrubs, as well as the effects of habitat differentiation. These findings will help to guide the restoration of degraded karst ecosystems, including the order of species establishment in different site types, as well as species selection and spatial distribution; they will also aid in the evaluation of restoration success. In general, topography has little effect on the diversity and size of trees in old-growth KFs. Future studies could explore the effects of soil and biological factors (e.g., seed dispersal and density dependence) on biodiversity, biomass, carbon, and mortality among sites.

## Data Availability Statement

The original contributions presented in the study are included in the article/supplementary material; further inquiries can be directed to the corresponding author.

## Author Contributions

JL drafted the manuscript. LZ analyzed data. YL conceived the idea and revised the manuscript. All authors contributed to the article and approved the submitted version.

## Funding

This paper was financially supported by the National Natural Science Foundation of China (nos. 32060340 and 31901309).

## Conflict of Interest

The authors declare that the research was conducted in the absence of any commercial or financial relationships that could be construed as a potential conflict of interest.

The reviewer YD declared a shared affiliation with the author LZ to the handling editor at the time of review.

## Publisher’s Note

All claims expressed in this article are solely those of the authors and do not necessarily represent those of their affiliated organizations, or those of the publisher, the editors and the reviewers. Any product that may be evaluated in this article, or claim that may be made by its manufacturer, is not guaranteed or endorsed by the publisher.

## Footnotes

^1^ https://www.esri.com/en-us/arcgis/

^2^ https://www.r-project.org/
